# Whole Genome Sequencing of Spontaneously Occurring Rat Natural Killer Large Granular Lymphocyte Leukemia Identifies JAK1 Somatic Activating Mutation

**DOI:** 10.3390/cancers12010126

**Published:** 2020-01-03

**Authors:** T. Tiffany Wang, Jun Yang, Shubha Dighe, Matthew W. Schmachtenberg, Nathan T. Leigh, Emily Farber, Suna Onengut-Gumuscu, David J. Feith, Aakrosh Ratan, Thomas P. Loughran, Thomas L. Olson

**Affiliations:** 1 Department of Medicine and University of Virginia Cancer Center, Division of Hematology & Oncology, University of Virginia School of Medicine, Charlottesville, VA 22908, USA; ttw2ws@virginia.edu (T.T.W.); jy5h@virginia.edu (J.Y.); sad6b@virginia.edu (S.D.); mws3v@virginia.edu (M.W.S.); nathangolf68@gmail.com (N.T.L.); djf2g@virginia.edu (D.J.F.);; 2 Center for Public Health Genomics, University of Virginia, Charlottesville, VA 22908, USA; ef8j@virginia.edu (E.F.); so4g@virginia.edu (S.O.-G.); ar7jq@virginia.edu (A.R.)

**Keywords:** JAK/STAT, leukemia, mutation, RNK-16, F344, NK cells, activation

## Abstract

Large granular lymphocyte (LGL) leukemia arises spontaneously in elderly Fischer (F344) rats. This rodent model has been shown to emulate many aspects of the natural killer (NK) variant of human LGL leukemia. Previous transplantation of leukemic material into young F344 rats resulted in several strains of rat NK (RNK) primary leukemic cells. One strain, RNK-16, was adapted into the RNK-16 cell line and established as an aggressive NK-LGL leukemia model. Whole genome sequencing of the RNK-16 cell line identified 255,838 locations where the RNK16 had an alternate allele that was different from F344, including a mutation in *Jak*1. Functional studies showed *Jak*1 Y1034C to be a somatic activating mutation that mediated increased STAT signaling, as assessed by phosphoprotein levels. Sanger sequencing of *Jak*1 in RNK-1, -3, -7, and -16 found only RNK-16 to harbor the Y1034C *Jak*1 mutation. In vivo studies revealed that rats engrafted with RNK-16 primary material developed leukemia more rapidly than those engrafted with RNK-1, -3, and -7. Additionally, ex vivo RNK-16 spleen cells from leukemic rats exhibited increased STAT1, STAT3, and STAT5 phosphorylation compared to other RNK strains. Therefore, we report and characterize a novel gain-of-function *Jak*1 mutation in a spontaneous LGL leukemia model that results in increased downstream STAT signaling.

## 1. Introduction

Large granular lymphocyte (LGL) leukemia is a lymphoproliferative neoplasm characterized by uncontrolled clonal expansion of LGLs of T-cell or natural killer (NK) cell origin, which causes anemia, neutropenia, and splenomegaly [1]. The World Health Organization classified LGL leukemia into three subtypes: T-cell LGL leukemia, chronic lymphoproliferative disorder of NK cells, and aggressive NK-cell leukemia [2]. The aggressive NK-cell leukemia is distinct from its indolent counterparts, affecting younger individuals with a higher prevalence in Asia and South America [3,4]. While the etiology of LGL leukemia is not well understood, LGL leukemia is characterized by multiple aberrant signaling pathways, including the Janus kinase/signal transducers and activators of the transcription (JAK/STAT) signaling pathway [1,5].

The JAK/STAT pathway relays extracellular signals from cytokines and other factors to intracellular factors to control nuclear transcription. This pathway is essential for various intracellular processes, including apoptosis, immune response, and differentiation. JAKs are non-receptor tyrosine kinases that interact with the intracellular tail of membrane-bound receptor complexes. JAK proteins consist of four domains; an N-terminal FERM (F for 4.1 protein, E for ezrin, R for radixin and M for moesin) domain, an SH2 domain, a pseudokinase domain, and a C-terminal protein tyrosine kinase (PTK) domain. Within the PTK domain lies an activation loop housing two highly conserved adjacent tyrosine residues. JAK signaling is well studied, and mutations in the JAK family are clearly implicated in the pathogenesis of hematological disorders [6,7]. JAK1 mutations have been reported in various hematological malignancies, including 7–27% of T-cell acute lymphoblastic leukemia (ALL) [8,9,10,11,12], 8–12% of T-cell prolymphocytic leukemia [13,14], 1.5% of B-ALL [9], and a small percentage of acute myeloid leukemia [15]. Subsequently, JAK1 mutations have been linked to solid tumor cancers, including Hepatitis B-associated hepatocellular carcinoma [16] and gynecological tumors [17].

JAK signaling phosphorylates downstream STAT proteins, important in regulating the transcription of genes involved in cell differentiation, survival, and proliferation [7,8]. Dysregulated STAT1, STAT3, and STAT5 signaling is well documented in LGL leukemia [18]. All patients exhibit constitutive STAT1 and STAT3 phosphorylation, and the occurrence of *STAT5B* [19,20,21] and *STAT3* [19,21,22,23] somatic activating mutations are a hallmark of LGL leukemia. Taken together, perturbations in the JAK/STAT pathway are well documented in hematological malignancies.

Fischer 344 rats are an inbred strain of rats that were used for decades as a primary in vivo model for cancer and toxicology studies [24]. Large granular lymphocyte (LGL) leukemia spontaneously occurs in 30–50% of Fischer 344 rats over 18 months of age [25,26,27]. Presentation of the disease in rats include anemia, splenomegaly, and leukemic cell infiltration of the spleen, liver, and lungs. [28]. Morphologically, these leukemic cells resemble LGLs with cytoplasmic granules. Functional studies using cytotoxicity and cell marker assays characterized these leukemic cells as being of NK cell origin and labelled as rat natural killer (RNK) cells [29]. Transplantation of primary RNK material induces leukemia, and has been utilized to establish 15 RNK strains. Of all strains, RNK-16 was characterized as a highly cytotoxic variant [29,30] and adapted into the RNK-16 cell line [31,32]. While high prevalence of disease and slight variations in features differ from the human counterpart, the morphological, functional, and clinical similarities established RNK-16 as a model of the human aggressive form of natural killer LGL leukemia (NK-LGL leukemia) [28,33,34].

The goal of the present study was to further characterize the RNK-16 cell line through whole genome sequencing (WGS). The results will aid interpretation of future LGL leukemia therapy response studies by our lab and others who use this model. Whole genome sequences from the RNK-16 cell line were compared to the F344 genome to reveal variants in several oncogenes. This led to the discovery of a novel *Jak*1 Y1034C mutation, which was also validated in ex vivo primary spleen material from leukemic rats. This allowed us to identify the mutated JAK1 as the putative leukemic driver JAK1, as well as mutations that occurred during adaptation to culture. Functional assays showed that *Jak1* Y1034C increased downstream STAT1, STAT3, and STAT5 phosphorylation in both the cell line and the primary RNK material. Characterization of this mutation may have a larger impact in future studies as more JAK1 mutations are uncovered in other diseases.

## 2. Results

### 2.1. Identification and Validation of Mutations in the RNK-16 Cell Line and RNK-16 Primary Spleen Material

**Figure 1:**
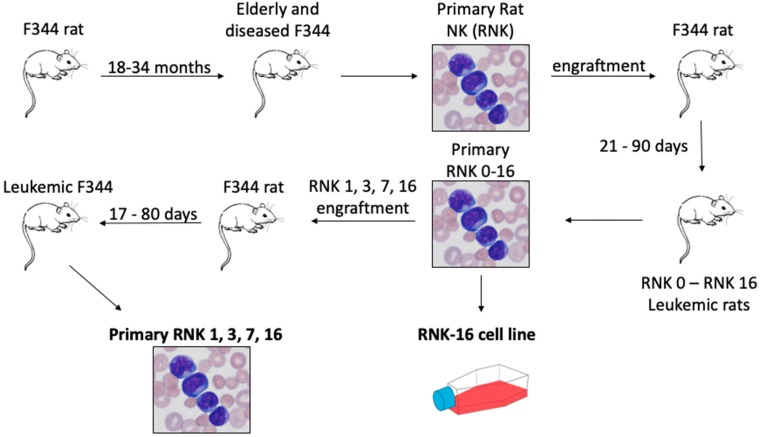
Diagram of rat natural killer (RNK) material. Aging F344 rats naturally develop LGL leukemia. Leukemic cells were transplanted back into young F344 rats to produce several strains of RNK [27]. RNK-16 cells were adapted into a cell line. Transplantation of RNK-1, -3, -7, and -16 into young F344 rats generated ex vivo spleen samples, and those samples in bold were used in these studies.

Whole genome sequencing (WGS) was successfully performed on the RNK-16 cell line in an effort to identify variants that contribute to NK cell leukemogenesis (Figure 1).

After filtering for quality control parameters and removing variants also seen in F344 genome, we were left with 255,838 variants (Table S1). Variants were annotated for their impact on the canonical transcripts using SnpEff. Restriction of genes with variants of a Phred quality score of over 20 and labelled as high or moderate impact resulted in 498 genes. Using the Homologene database, these 498 genes were mapped to 457 human genes, of which 157 did not have the same names (Table S2). Those with matched gene names were queried in the COSMIC Cancer Gene Census, which classifies genes into two tiers based on the extent of evidence that variants in those genes drive oncogenic transformation [35]. Eight genes with mutations in RNK-16 were labelled as having oncogenic transformative potential, including *Brd*3, *Ddr*2, *Fat*1, *Jak*1, *Ncor*2 *Nono*, *Stag*, and *Thrap*3 (Table 1).

**Figure 2:**
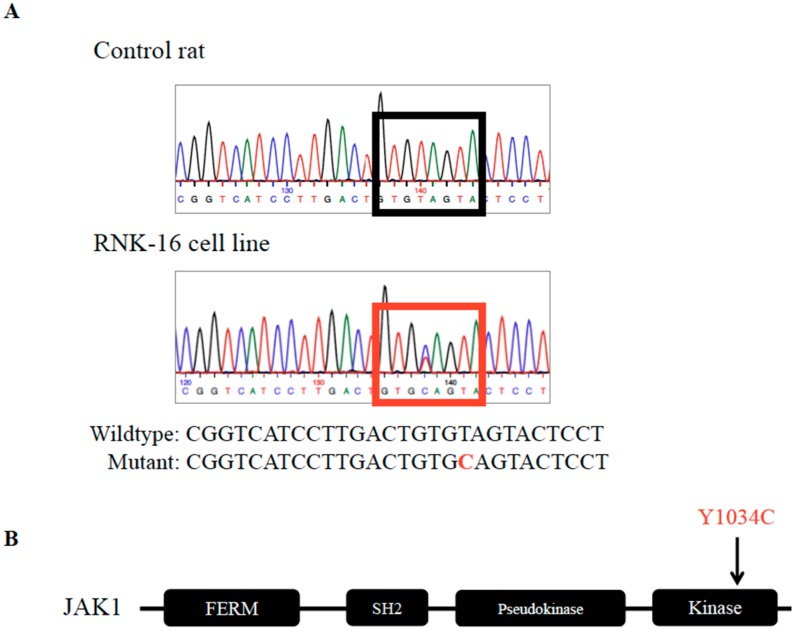
Validation of JAK1 variant by Sanger sequencing. (**A**) Sequencing chromatographs from Sanger sequencing validate JAK1 mutation in RNK-16 cell line. (**B**) JAK1 variant occurs in the kinase domain at Y1034, resulting in a change from tyrosine to cysteine. It sits within the activation loop, adjacent to another phosphorylation site at Y1033.

Sequencing strategies were designed to validate the variants by Sanger sequencing in the RNK-16 cell line. Out of the eight genes, variants were confirmed in five, including *Ddr2*, *Fat1*, *Jak1*, *Ncor2*, and *Stag*. As the RNK-16 cell line was adapted to an in vitro culture from ex vivo RNK-16 material, validation of these mutations was necessary to demonstrate that mutations were not the result of cell line transformation. Sanger sequencing of ex vivo RNK-16 spleen cells (Figure 1) confirmed only the *Jak*1 variant (Figure 2A), indicating that the adaptation of RNK-16 primary cells into the RNK-16 cell line may have resulted in the gain of mutations in *Ddr2*, *Fat1*, *Ncor2*, and *Stag*, but not *Jak1*. JAK1 is a tyrosine kinase involved in cytokine signaling that results in the downstream activation of STATs. This *Jak1* mutation is found in the PTK domain and is located within the activation loop that contains two tyrosine phosphorylation sites, 1033 and 1034 (Figure 2B). Results of WGS, as well as Sanger validation of the cell line and primary splenocyte material, are summarized in Table 1.

### 2.2. JAK1 Mutation Increased Downstream STAT Signaling

**Figure 3:**
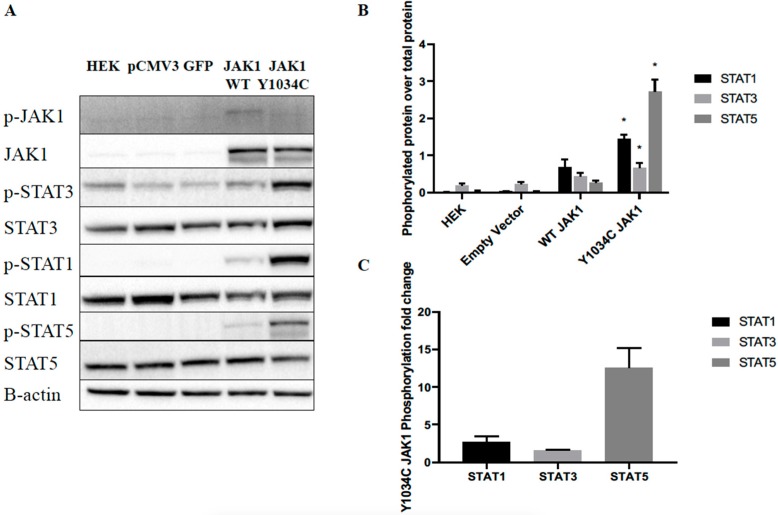
*Jak1* Y1034C mutation increased STAT protein phosphorylation in HEK293FT cells. HEK293FT cells were transfected with pCMV3 vectors containing no insert, GFP, WT rat JAK1, or rat JAK1 Y1034C. At 72 h post transfection, cells were harvested, and protein lysates were analyzed by western blotting. Membranes were probed with the phosphorylated and total antibodies of JAK1, STAT3, STAT1, and STAT5. A representative western blot shown in (**A**), quantification of all replicates (*n* = 4) with SEM is graphed in (**B**), and the calculation of fold change in JAK1 Y1034C phosphorylated proteins over WT JAK1 phosphorylated proteins from all replicates with SEM is graphed in (**C**). Statistics were performed for mutant JAK1 relative to WT JAK1. * = *p* < 0.05. More details of western blot, please view at the supplementary materials.

To examine the effects of Y1034C *Jak1* mutation on protein function, we transiently expressed wild-type (WT) *Jak1* or *Jak1* Y1034C in HEK293-FT cells. Western blotting demonstrated constitutive phosphorylation of downstream STAT targets, including STAT1, STAT3, and STAT5 (Figure 3). The epitope of the phosphorylated JAK1 antibody recognizes the dual tyrosine phosphorylation in the activation loop (1033/1034), which is altered in the mutant; therefore, it was unable to recognize the mutant phosphorylated protein, even though mutant overexpression was confirmed by the total JAK1 antibody. The total JAK1 protein levels were unchanged, indicating that mutation did not increase production of total JAK1. Low levels of all phosphoprotein targets were observed when transfected with WT *Jak1*, drastically different from the large increase of phosphorylation with the transfection of *Jak1* Y1034C. Thus, insertion of *Jak1* Y1034C induced the constitutive activation of STAT signaling, independent of exogenous cytokine signaling.

### 2.3. In Vivo and Ex Vivo Characterization of Primary Rat Natural Killer Material Shows an Aggressive Disease Course and Increased STAT Signaling With Mutant JAK1

**Figure 4:**
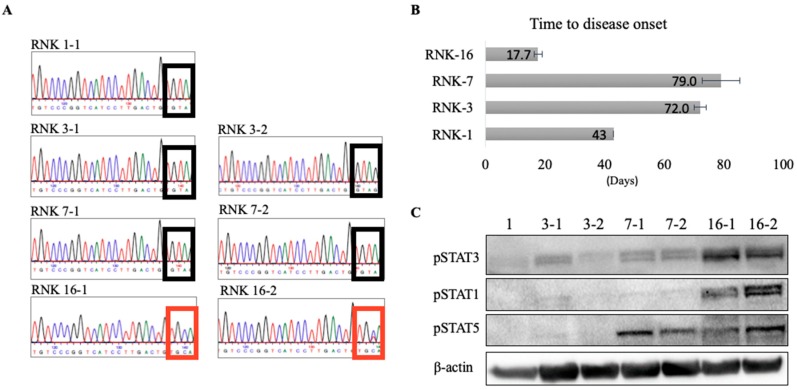
In vivo and ex vivo characterization of RNK material. A total of 1 x 10^6^ cells from RNK-1, -3, -7, and -16 were injected i.p. into young F344 rats and the time to disease was measured. (**A**) Sanger sequencing of primary spleen mononuclear cells harvested from leukemic animals showed a *Jak*1 Y1034C mutation found only in RNK-16. (**B**) Those engrafted with RNK-16 had significantly shorter time to disease onset compared to the others. (**C**) Spleen cells were harvested from leukemic rats at euthanasia. Immunoblot analysis showed increased STAT1, -3, and -5 phosphorylation in RNK-16 relative to RNK-1, -3, and -7. More details of western blot, please view at the supplementary materials.

Previous characterization of primary ex vivo RNK material reported RNK-16 to have high cytotoxicity compared to RNK-1 and RNK-7 [29,30]. Characterization of RNK-3 was not previously published, although all of the RNK material was isolated together. Additionally, our Sanger sequencing of ex vivo RNK-1, -3, -7, and -16 spleen material detected *Jak1* Y1034C only in RNK-16 (Figure 4A). To further characterize these RNK strains, one million viable primary RNK-1, -3, -7, and -16 cells were injected intraperitoneally (i.p.) into young F344 rats to study leukemia onset of RNK-16 compared to other strains. Rats engrafted with RNK-16 showed significantly shorter time to disease onset, as determined by absolute white blood count and weight loss (Figure 4B). The average time to disease onset for RNK-16 was 17.7 days, less than half the time for rats injected with RNK-1 to develop leukemia and less than a quarter of the time for rats transplanted with RNK-3 and RNK-7 to develop disease. Protein extracts from spleen cells harvested from leukemic rats were run on western blots and probed for downstream STAT phosphorylation (Figure 4C). RNK-16 spleen cells had increased STAT1 and STAT3 phosphorylation relative to all other RNKs, as well as similar levels of STAT5 phosphorylation to RNK-7. Taken together, these studies provide molecular and biochemical data to complement the previously published functional characterization of RNK primary material.

## 3. Discussion

This study discovered and characterized a *Jak1* Y1034C mutation found in a naturally occurring F344 rat NK-LGL leukemia model. RNK-16, characterized as highly cytotoxic [30], was established as an in vitro cell line [32] and model for aggressive NK-LGL leukemia [34]. Whole genome sequencing in the RNK-16 cell line revealed variants in eight oncogenes. Sanger sequencing of the RNK-16 cell line and ex vivo RNK-16 spleen material confirmed a single variant, *Jak1* Y1034C, in all tested material. Lack of validation of other variants found in the RNK-16 cell line in the ex vivo RNK-16 spleen material indicates that mutations may have been acquired as a result of in vitro cell culture expansion [36].

Aberrant activation of JAK/STAT signaling is a hallmark of LGL leukemia. Increased activation of this pathway results from increased cytokine signaling [4,37] and somatic activating mutations in STAT3 [19,22,38] and STAT5B [19,20,39], further implicating the oncogenic role of this pathway in LGL leukemia. WGS on a small cohort of NK-LGL leukemia patients did not reveal mutations in *JAK1* (manuscript under review). While *JAK1* mutations have not been reported in LGL leukemia patients, mutations in *JAK1* have been identified in several hematological malignancies [9,10,11,13,15,40]. The majority of mutations occur in the pseudokinase domain, and have been characterized to have oncogenic potential [41]; patients with such mutations have poor prognoses [40].

The activation loop within the C-terminal PTK domain includes two adjacent tyrosine phosphorylation sites. Mutation of the first tyrosine to an alanine abrogated kinase activation, indicating that the first tyrosine is necessary for JAK1 activation. An alanine substitution to the second tyrosine allowed kinase activation, showing that activation is not dependent upon the second tyrosine [42]. We detected a mutation at Y1034, which is the second tyrosine within the activation loop. While not necessary for kinase activation, mutations at this location may affect enzymatic activity [43], as well as the specificity and strength of interactions with substrates, thereby leading to altered downstream signaling. Random mutagenesis experiments on human *JAK1* have shown that the specific substitution of cysteine for the second tyrosine, analogous to the mutation that we detected in rat JAK1, drives clonal outgrowth and increases JAK/STAT signaling [41].

Here we demonstrated that *Jak1* Y1034C is a naturally occurring somatic mutation found in leukemic rats that was shown to elevate the phosphorylated levels of STAT1, STAT3 and STAT5. We observed the largest fold change of phosphorylated STAT5 by mutant relative to WT JAK1, suggesting STAT5 may be more readily phosphorylated by mutant JAK1 [42,44]. Mutation of JAK1 did not increase total JAK1 protein. While we were unable to assess the phosphorylation of JAK1 itself, presumably due to the overlap of mutation location and antibody epitope, increased phosphorylation of JAK1 was shown when the substitution of cysteine for the second tyrosine was performed in human *JAK1* [42]. Additionally, future mass spectrometry-based analyses could be insightful in this regard.

In vivo studies involved the transplantation of primary RNK-1, -3, -7, and -16 material into young F344 rats. Previous characterization of these strains specified high cytotoxicity of RNK-16 compared to other strains, and our study enhanced their findings by examining time to disease onset, JAK1 mutation status, and phosphorylated STAT protein levels. We found that rats transplanted with RNK-16 have significantly more rapid disease development. The *Jak1* Y1034C mutation was detected only in ex vivo RNK-16 spleen material, which also exhibited increased protein levels of phosphorylated STAT1, STAT3 and STAT5. High cytotoxicity of RNK-16 compared to other RNK strains may be in part explained by the *Jak1* Y1034C mutation, which increases downstream STAT signaling and may drive more aggressive leukemogenesis. Interestingly, we observed increased phosphorylated STAT5 in ex vivo RNK-7 material, suggesting that other factors aside from a *Jak1* mutation may promote STAT5 phosphorylation. Differing genetic, epigenetic, and signaling alterations may explain differences between the various RNK strains. Performing WGS on other RNK primary material may reveal other variants in addition to the *Jak1* Y1034C mutation that may contribute to the pathogenesis of rat NK-LGL leukemia.

## 4. Materials and Methods

### 4.1. Acquisition of Sequencing Data

Paired-end Illumina short read sequences were downloaded for the F344/NCrl sample from the European Nucleotide Archive (ENA; Run: ERR224448). The RNK-16 cell line was kindly provided by Drs. Craig Reynolds and Howard Young (National Cancer Institute, Bethesda, MD, USA). DNA was isolated by AnaPrep (BioChain Institute, Newark, CA, USA) and quantified by Qubit (Thermo Fisher (Waltham, MA, USA). A sequencing library was prepared with a Nextera (San Diego, CA, USA) kit, using half the recommended DNA input and paired-end sequenced on the NextSeq 500 (Illumina (San Diego, CA, USA)) at the Center for Public Health Genomics at the University of Virginia.

### 4.2. Alignments

Illumina short-read sequences from the RNK-16 cell line and the F344 sample were aligned to the *Rattus norvegicus* reference sequence (rn6) with BWA mem [45], using the default parameters. Putative PCR duplicates were flagged using SAMBLASTER [46], which was also used to add MC and MQ tags to all the output paired-end alignments, and separate the split-reads, the discordant pairs, and the unmapped sequences from the resulting output (Table S3). The SAM outputs were converted to BAM format and sorted by chromosomal coordinates using SAMTools [47]. All BAM files for the same samples were merged using SAMTools, and BAMreport (https://github.com/aakrosh/BAMreport) was then used to generate the alignment statistics and metrics.

### 4.3. CNV Map

Evidence of GC bias were found in both sequence datasets (Table S3), and the same diagnostic plots also revealed evidence of large copy number aberrations in the RNK-16 sample that could confound variant calls. From there, a copy number variant map was created to define the non-uniform ploidy variation in the RNK-16 sample by binning the aligned fragments into intervals of 10 kb mappable bases. Then, a locally weighted linear regression modeled the relationship between GC content and coverage in those intervals. This LOWESS fit was then used to scale the coverage in each bin, based on the mean coverage in bins with the same GC content. Circular binary segmentation (CBS) [48] identified bins that shared the same copy number state, and a Gaussian mixture model was used to genotype the segments and calculate their copy number state.

### 4.4. Identification of Variants

FreeBayes [49] identified the variants in the samples, requiring that the allele fraction in at least one sample be 0.1 and supported by two reads. The CNV map was provided as an input, and required that the Phred scaled probability of a polymorphism at the loci be greater than 5. The following additional options were used: —pooled-discrete, —pooled-continuous, —genotype-qualities, —report-genotype-likelihood-max and —allele-balance-priors-off.

### 4.5. Annotation and Filtering of Variants

Variants where the F344/NCrl sample was homozygous for the reference were labelled, and an alternate allele in RNK-16 was observed as somatic, using filters implemented similar to SpeedSeq [50]. To start, genotype likelihoods were extracted from the output of FreeBayes. For RNK-16, we retrieved the best likelihood to not be the reference, and for F344/NCrl, the best likelihood was to be the reference. We required that (a) both the retrieved likelihoods be above a minimum threshold, and (b) we filtered to remove any low-frequency variants in RNK-16 that were also present in the F344/NCrl sample at low frequencies. We then used SnpEff [51] to annotate these labeled variants, using dbSnp and the variant calls from Hermsen et al. [52]. This left us with 255,838 positions, with evidence of an alternate allele in RNK-16 (Table S1).

### 4.6. Gene Enrichment Analyses

Genes with variants were filtered for a Z score of >20 and those labelled with a medium or high impact. Homolog analysis of these gene variants was performed using DAVID (Database for Annotation, Visualization, and Integrated Discovery), resulting in a list of 458 human genes, of which 157 did not have the same name (Table S2). Variant genes were queried in the CGC (Cancer Gene Census) to determine which were implicated in cancers.

### 4.7. Sanger Sequencing to Validate Whole Genome Sequencing Results

Sequencing primers from Eurofins for eight oncogenes identified in COSMIC were designed using Primerblast [53] (Table S4). Bulk DNA from RNK-1, -3, -7, and -16 was sent to Eurofins for Sanger sequencing to determine oncogene mutation status.

### 4.8. JAK1 Mutagenesis

A full-length clone of the rat JAK1 sequence (XM_006238453.1) in the pCMV3 vector was purchased from Sinobiological (Cat. RG81640-UT), as was the pCMV3 empty vector (Cat. CV011) and pCMV3–GFP (Green Florescent Protein) control vector (Cat. CV026). A JAK1 Y1034C mutation was introduced into the rat JAK1 pCMV3 using an In-Fusion HD Site-directed Mutagenesis kit (Takara (Kusatsu, Shiga Prefecture, Japan), Cat. 638909), with primers acquired from Eurofins (F: 5’ AGACCGATAAGGAGTACTACAGT 3’, R: 5’ GGAGTACTACACAGTCAAGGATGAC 3’). Primers were designed to overlap by 15 bp at the 5’, ends with a GC content of 40–50%. The mutant DNA was transformed into Stellar Competent cells, and plasmid DNA was isolated. Insertion of only the desired Y1034C mutation and presence of a full-length JAK1 were confirmed by Sanger sequencing.

### 4.9. Cell Culture

RNK-16 cell line (kindly provided by Dr. Craig Reynolds, NCI) was cultured in RPMI 1640 supplemented with 10% Fetal Bovine Serum (FBS), non-essential amino acids (Sigma (St. Louis, MO, USA)), 1% sodium pyruvate (Sigma), and 25 μM 2-mercaptoethanol. HEK293-FT (human embryonic kidney) cells (kindly provided by Kenichiro Doi and H.G Wang, PSU College of Medicine) were cultured in DMEM with 10% FBS.

### 4.10. Transfection and Western Blot Analysis

The empty vector, GFP, WT JAK1, or mutant JAK1 cDNA was transfected using Lipofectamine 3000 (Invitrogen (Carlsbad, CA, USA), Cat. L3000008) into HEK293-FT cells. High transfection efficiency was determined via GFP transfection control. Whole cell lysates were prepared in RIPA buffer (ThermoFisher (Carlsbad, CA, USA)) 72 h post-transfection; proteins were electrophoresed on a 4–12% acrylamide gel (ThermoFisher) and subsequently transferred to a Polyvinylidene fluoride (PVDF) membrane. Membranes were blocked and incubated with varying antibodies. Cell signaling technology primary antibodies used in this study included Phospho-STAT3 (Y705, Cat #9131), STAT3 (Cat #9139), Phospho-STAT1 (Y701, Cat #&649), STAT1 (CAT #9175), Phospho-STAT5 (Y694, Cat #9351), STAT5 (Cat #9363), Phospho-JAK1 (Y1034/1035, Cat #3331), and JAK1 (Cat# 3344). After incubation overnight at 4 °C with a primary antibody, blots were incubated with a secondary antibody (anti-rabbit IgG–HRP (Horseradish Peroxidase) linked (Cat #7073) or anti-mouse IgG–HRP linked (Cat #7076)) for one hour, and washed before treating with Clarity enhanced chemiluminescence (ECT; Biorad) reagent. Membranes were imaged using a ChemiDoc MP instrument and analyzed using ImageLab (Biorad (Hercules, CA, USA)).

### 4.11. Animal Study

Animal experimentation was performed according to protocols approved by the Institutional Animal Care and Use Committee at the University of Virginia (Protocol # 4014-05-17). Male F344 rats of approximately 5 weeks of age were obtained from Charles River Laboratory. After one week of conditioning to our vivarium, one million viable RNK-1, RNK-3, RNK-7, or RNK-16 primary cells were transplanted intraperitoneally into F344 rats in triplicate for all groups, except for RNK-1, where there were not enough viable cells to engraft three animals. Peripheral blood samples were collected weekly to monitor leukemia development after RNK cell engraftment. Rats losing 20% body weight most likely developed NK-LGL leukemia and were euthanized. At necropsy, rat spleens were harvested. Splenocytes were isolated using s Ficoll–Hypaque density gradient centrifugation and cryopreserved in liquid nitrogen for future use. Rat blood was collected using Becton Dickinson (BD) (Franklin Lakes, NJ, USA) microcontainers with K2EDTA (BD 365974). Blood smears were prepared using a standard technique, and staining was done using a VWR (Radnor, PA, USA) Hematology Quick stain kit (VWR#10143-224). Slides were examined (Olympus (Shinjuku, Tokyo, Japan) BX51) using immersion oil. Frozen splenocytes were taken from liquid nitrogen, and protein harvested for western blotting analysis, as described above.

### 4.12. Statistical Analysis

Statistical analyses for all in vitro systems utilized unpaired Student’s *t*-test, with *p* < 0.05 considered statistically significant. Analyses were performed in both Microsoft Excel and GraphPad Prism.

## 5. Conclusions

In summary, this work presents WGS of a spontaneously occurring NK-LGL leukemia model, leading to the discovery and characterization of a somatic activating *Jak1* Y1034C mutation. The activating mutant may be a useful tool for biochemical studies of JAK/STAT signaling. This work reinforces the critical contributions of JAK/STAT pathway activation in hematologic malignancies, especially those of T- and NK-cell origin. Additionally, RNK-16 engraftment represents an in vivo model of a leukemia driven by mutationally activated JAK1, which may have utility for the evaluation and development of JAK1 inhibitors.

## Figures and Tables

**Table 1:** Cancer Gene Census (CGC) query revealed genes mutated in the RNK-16 cell line that are implicated in cancer. Tier levels are assigned by CGC and indicate a depth of evidence of mutations in those genes having oncogenic transformative potential. Variants in *Ddr*2, *Fat*1, *Jak*1, *Ncor*2, and *Stag* were confirmed using Sanger sequencing in the RNK-16 cell line. Only the mutation in *Jak*1 was confirmed in the RNK-16 primary spleen cells.

Gene Symbol	Tier Level	RNK-16 Cell Line WGS	RNK-16 Cell Line Validation	RNK-16 Primary Spleen Cells
*Brd3*	1	✓	x	x
*Ddr2*	1	✓	✓	x
*Fat1*	1	✓	✓	x
*Jak1*	1	✓	✓	✓
*Ncor2*	1	✓	✓	x
*Nono*	1	✓	x	x
*Stag*	1	✓	✓	x
*Thrap3*	1	✓	x	x

## Supplementary Materials

The following are available online at https://www.mdpi.com/2072-6694/12/1/126/s1. Table S1: Alignment statistics for the RNK-16 and F344/NCrl samples, Table S2: Complete list of variants with an alternate allele in the RNK-16 cell line compared to F344/NCrl, Table S3: List of human homolog genes mapped to rat genes, with variants in RNK-16 cell line filtered by Q score and impact factor, Table S4: Primer design for WGS validation by Sanger sequencing.

## References

[1] Watters R.J., Liu X., Loughran T.P. (2011). T-cell and natural killer-cell large granular lymphocyte leukemia neoplasias. Leuk. Lymphoma.

[2] Swerdlow S.H., Campo E., Pileri S.A., Harris N.L., Stein H., Siebert R., Advani R., Ghielmini M., Salles G.A., Zelenetz A.D. (2016). The 2016 revision of the World Health Organization classification of lymphoid neoplasms. Blood.

[3] Suzuki R., Suzumiya J., Nakamura S., Aoki S., Notoya A., Ozaki S., Gondo H., Hino N., Mori H., Sugimori H. (2004). Aggressive natural killer-cell leukemia revisited: Large granular lymphocyte leukemia of cytotoxic NK cells. Leukemia.

[4] Lamy T., Moignet A., Loughran T.P. (2017). LGL leukemia: From pathogenesis to treatment. Blood.

[5] Steinway S.N., Leblanc F., Loughran T.P. (2014). The pathogenesis and treatment of large granular lymphocyte leukemia. Blood Rev..

[6] Hammarén H.M., Virtanen A.T., Raivola J., Silvennoinen O. (2019). The regulation of JAKs in cytokine signaling and its breakdown in disease. Cytokine.

[7] Ward A.C., Touw I., Yoshimura A. (2000). The Jak-Stat pathway in normal and perturbed hematopoiesis. Blood.

[8] Vainchenker W., Constantinescu S.N. (2013). JAK/STAT signaling in hematological malignancies. Oncogene.

[9] Li Q., Li B., Hu L., Ning H., Jiang M., Wang D., Liu T., Zhang B., Chen H. (2017). Identification of a novel functional JAK1 S646P mutation in acute lymphoblastic leukemia. Oncotarget.

[10] Flex E., Petrangeli V., Stella L., Chiaretti S., Hornakova T., Knoops L., Ariola C., Fodale V., Clappier E., Paoloni F. (2008). Somatically acquired JAK1 mutations in adult acute lymphoblastic leukemia. J. Exp. Med..

[11] Jeong E.G., Kim M.S., Nam H.K., Min C.K., Lee S., Chung Y.J., Yoo N.J., Lee S.H. (2008). Somatic mutations of JAK1 and JAK3 in acute leukemias and solid cancers. Clin. Cancer Res..

[12] Zhang J., Ding L., Holmfeldt L., Wu G., Heatley S.L., Payne-Turner D., Easton J., Chen X., Wang J., Rusch M. (2012). The genetic basis of early T-cell precursor acute lymphoblastic leukaemia. Nature.

[13] Bellanger D., Jacquemin V., Chopin M., Pierron G., Bernard O.A., Ghysdael J., Stern M.-H. (2014). Recurrent JAK1 and JAK3 somatic mutations in T-cell prolymphocytic leukemia. Leukemia.

[14] Kiel M.J., Velusamy T., Rolland D., Sahasrabuddhe A.A., Chung F., Bailey N.G., Schrader A., Li B., Li J.Z., Ozel A.B. (2014). Integrated genomic sequencing reveals mutational landscape of T-cell prolymphocytic leukemia. Blood.

[15] Xiang Z., Zhao Y., Mitaksov V., Fremont D.H., Kasai Y., Molitoris A., Ries R.E., Miner T.L., McLellan M.D., DiPersio J.F. (2008). Identification of somatic JAK1 mutations in patients with acute myeloid leukemia. Blood.

[16] Kan Z., Zheng H., Liu X., Li S., Barber T.D., Gong Z., Gao H., Hao H., Willard M.D., Xu J. (2013). Whole-genome sequencing identifies recurrent mutations in hepatocellular carcinoma. Genome Res..

[17] Ren Y., Zhang Y., Liu R.Z., Fenstermacher D.A., Wright K.L., Teer J.K., Wu J. (2013). JAK1 truncating mutations in gynecologic cancer define new role of cancer-associated protein tyrosine kinase aberrations. Sci. Rep..

[18] Epling-Burnette P.K., Liu J.H., Catlett-Falcone R., Turkson J., Oshiro M., Kothapalli R., Li Y., Wang J.-M., Yang-Yen H.-F., Karras J. (2001). Inhibition of STAT3 signaling leads to apoptosis of leukemic large granular lymphocytes and decreased Mcl-1 expression. J. Clin. Investig..

[19] Küçük C., Jiang B., Hu X., Zhang W., Chan J.K.C., Xiao W., Lack N., Alkan C., Williams J.C., Avery J.N. (2015). Activating mutations of STAT5B and STAT3 in lymphomas derived from γδ-T or NK cells. Nat. Commun.

[20] Andersson E.I., Tanahashi T., Sekiguchi N., Gasparini V.R., Bortoluzzi S., Kawakami T., Matsuda K., Mitsui T., Eldfors S., Bortoluzzi S. (2016). High incidence of activating STAT5B mutations in CD4-positive T-cell large granular lymphocyte leukemia. Blood.

[21] Rajala H.L.M., Porkka K., Maciejewski J.P., Loughran T.P., Mustjoki S. (2014). Uncovering the pathogenesis of large granular lymphocytic leukemia-novel STAT3 and STAT5b mutations. Ann. Med..

[22] Koskela H.L.M., Eldfors S., Ellonen P., van Adrichem A.J., Kuusanmäki H., Andersson E.I., Lagström S., Clemente M.J., Olson T., Jalkanen S.E. (2012). Somatic STAT3 mutations in large granular lymphocytic leukemia. N. Engl. J. Med..

[23] Andersson E., Kuusanmäki H., Bortoluzzi S., Lagström S., Parsons A., Rajala H., van Adrichem A., Eldfors S., Olson T., Clemente M.J. (2016). Activating somatic mutations outside the SH2-domain of STAT3 in LGL leukemia. Leukemia.

[24] Maronpot R.R., Nyska A., Foreman J.E., Ramot Y. (2016). The legacy of the F344 rat as a cancer bioassay model (a retrospective summary of three common F344 rat neoplasms). Crit. Rev. Toxicol..

[25] Stromberg P.C. (1985). Large granular lymphocyte leukemia in F344 rats. Model for human T gamma lymphoma, malignant histiocytosis, and T-cell chronic lymphocytic leukemia. Am. J. Pathol..

[26] Reynolds C.W., Timonen T., Herberman R.B. (1981). Natural killer (NK) cell activity in the rat. I. Isolation and characterization of the effector cells. J. Immunol..

[27] Losco P.E., Ward J.M. (1984). The Early Stage of Large Granular Lymphocyte Leukemia in the F344 Rat. Vet. Pathol..

[28] Thomas J., Haseman J.K., Goodman J.I., Ward J.M., Loughran T.P., Spencer P.J. (2007). A review of large granular lymphocytic leukemia in fischer 344 Rats as an initial step toward evaluating the implication of the endpoint to human cancer risk assessment. Toxicol. Sci..

[29] Ward J.M., Reynolds C.W. (1983). Large granular lymphocyte leukemia. A heterogeneous lymphocytic leukemia in F344 rats. Am. J. Pathol..

[30] Reynolds C.W., Bere E.W., Ward J.M. (1984). Natural killer activity in the rat. III. Characterization of transplantable large granular lymphocyte (LGL) leukemias in the F344 rat. J. Immunol..

[31] Ryan J.C., Niemi E.C., Nakamura M.C. (2000). Functional analysis of natural killer cell receptors in the RNK-16 rat leukemic cell line. Methods Mol. Biol..

[32] Axberg I., Nose M., Reynolds C.W., Wigzell H. (1988). Features of the in vitro established rat large granular lymphocyte leukaemia RNK-16. Scand. J. Immunol..

[33] Reynolds C.W., Foon K.A. (1984). T gamma-lymphoproliferative disease and related disorders in humans and experimental animals: A review of the clinical, cellular, and functional characteristics. Blood.

[34] Ishmael J., Dugard P.H. (2006). A review of perchloroethylene and rat mononuclear cell leukemia. Regul. Toxicol. Pharmacol..

[35] Sondka Z., Bamford S., Cole C.G., Ward S.A., Dunham I., Forbes S.A. (2018). The COSMIC cancer gene census: Describing genetic dysfunction across all human cancers. Nat. Rev. Cancer.

[36] Kim M., Rhee J.-K., Choi H., Kwon A., Kim J., Lee G.D., Jekarl D.W., Lee S., Kim Y., Kim T.-M. (2017). Passage-Dependent Accumulation Of Somatic Mutations In Mesenchymal Stromal Cells During In Vitro Culture Revealed By Whole Genome Sequencing. Sci. Rep..

[37] Coppe A., Andersson E.I., Binatti A., Gasparini V.R., Bortoluzzi S., Clemente M., Herling M., Maciejewski J., Mustjoki S., Bortoluzzi S. (2017). Genomic landscape characterization of large granular lymphocyte leukemia with a systems genetics approach. Leukemia.

[38] Andersson E.I., Rajala H.L.M., Eldfors S., Ellonen P., Olson T., Jerez A., Clemente M.J., Kallioniemi O., Porkka K., Heckman C. (2013). Novel somatic mutations in large granular lymphocytic leukemia affecting the STAT-pathway and T-cell activation. Blood Cancer J..

[39] Klein K., Witalisz-Siepracka A., Maurer B., Prinz D., Heller G., Leidenfrost N., Prchal-Murphy M., Suske T., Moriggl R., Sexl V. (2019). STAT5B N642H drives transformation of NKT cells: A novel mouse model for CD56 + T-LGL leukemia. Leukemia.

[40] Arulogun S.O., Choong H.-L., Taylor D., Ambrosoli P., Magor G., Irving I.M., Tee-Beng Keng T.-P., Perkins A.C. (2017). JAK1 somatic mutation in a myeloproliferative neoplasm. Haematologica.

[41] Gordon G.M., Lambert Q.T., Daniel K.G., Reuther G.W. (2010). Transforming JAK1 mutations exhibit differential signalling, FERM domain requirements and growth responses to interferon-γ. Biochem. J..

[42] Liu K.D., Gaffen S.L., Goldsmith M.A., Greene W.C. (1997). Janus kinases in interleukin-2-mediated signaling: JAK1 and JAK3 are differentially regulated by tyrosine phosphorylation. Curr. Biol..

[43] Haan S., Margue C., Engrand A., Rolvering C., de Leur H.S.-V., Heinrich P.C., Behrmann I., Haan V. (2008). Dual role of the Jak1 FERM and kinase domains in cytokine receptor binding and in stimulation-dependent Jak Activation. J. Immunol..

[44] Witalisz-Siepracka A., Klein K., Prinz D., Leidenfrost N., Schabbauer G., Dohnal A., Sexl V. (2018). Loss of JAK1 drives innate immune deficiency. Front. Immunol..

[45] Li H. (2013). Aligning Sequence Reads, Clone Sequences and Assembly Contigs with BWA-MEM. arXiv.

[46] Faust G.G., Hall I.M. (2014). SAMBLASTER: Fast duplicate marking and structural variant read extraction. Bioinformatics.

[47] Li H., Handsaker B., Wysoker A., Fennell T., Ruan J., Homer N., Marth G., Abecasis G., Durbin R., 1000 Genome Project Data Processing Subgroup (2009). The Sequence Alignment/Map format and SAMtools. Bioinformatics.

[48] Olshen A.B., Venkatraman E.S., Lucito R., Wigler M. (2004). Circular binary segmentation for the analysis of array-based DNA copy number data. Biostatistics.

[49] Garrison E., Marth G. (2012). Haplotype-Based Variant Detection from Short-Read Sequencing. arXiv.

[50] Chiang C., Layer R.M., Faust G.G., Lindberg M.R., Rose D.B., Garrison E.P., Marth G.T., Quinlan A.R., Hall I.M. (2015). SpeedSeq: Ultra-fast personal genome analysis and interpretation. Nat. Methods.

[51] Cingolani P., Platts A., Wang L.L., Coon M., Nguyen T., Wang L., Land S.J., Lu X., Ruden D.M. (2012). A program for annotating and predicting the effects of single nucleotide polymorphisms, SnpEff: SNPs in the genome of Drosophila melanogaster strain w1118; iso-2; iso-3. Fly (Austin).

[52] Hermsen R., de Ligt J., Spee W., Blokzijl F., Schäfer S., Adami E., Boymans S., Flink S., van Boxtel R., van der Weide R.H. (2015). Genomic landscape of rat strain and substrain variation. BMC Genom..

[53] Ye J., Coulouris G., Zaretskaya I., Cutcutache I., Rozen S., Madden T.L. (2012). Primer-BLAST: A tool to design target-specific primers for polymerase chain reaction. BMC Bioinform..

