# Supplementary material for: Whole Genome Sequencing of Spontaneously Occurring Rat Natural Killer Large Granular Lymphocyte Leukemia Identifies JAK1 Somatic Activating Mutation
Source: Cancers (Basel). 2020 Jan 3;12(1):126. doi: 10.3390/cancers12010126 (PMC7017127; doi:10.3390/cancers12010126)
Supplement: Supplementary file 1 [file cancers-12-00126-s001.zip › cancers-656994-english-supplementary/cancers-656994-english-supplementary-.docx]

Supplementary Materials

Whole Genome Sequencing of Spontaneously Occurring Rat Natural Killer Large Granular Lymphocyte Leukemia Identifies JAK1 Somatic Activating Mutation

T. Tiffany Wang, Jun Yang, Shubha Dighe, Matthew W. Schmachtenberg, Nathan T. Leigh, Emily Farber, Suna Onengut-Gumuscu, David J. Feith, Aakrosh Ratan, Thomas P. Loughran, Jr., Thomas L. Olson

**Table S1.** Alignment statistics for the RNK16 and F344/NCrl samples.

| **Sample** | **Reads** | **Aligned (%)** | **Duplicates (%)** | **Properly paired (%)** |
| --- | --- | --- | --- | --- |
| **RNK-16** | 657,092,472 | 96.02 | 2.73 | 93.46 |
| **F344/NCrl** | 807,260,796 | 99.18 | 5.55 | 95.59 |

**Table S2–S3.** Please view at the excel file.

**Table S4.** Primer design for WGS validation by Sanger sequencing.

| **Primer** | **Sequencing Range** | | **Start site** | **5'- sequence-3'** | **Tm (˚C)** | **Length** | **GC Content** |
| --- | --- | --- | --- | --- | --- | --- | --- |
| FOR_1 | 701 | 1400 | 654 | AGATGCAGTTGCCAGAGCTT | 60.164 | 20 | 50 |
| FOR_2 | 1401 | 2100 | 1347 | TACAGAATGGCTGTCACGGT | 59.165 | 20 | 50 |
| FOR_3 | 2101 | 2800 | 2056 | GCCATTGGATCTCTTCATGC | 60.579 | 20 | 50 |
| FOR_4 | 2801 | 3500 | 2756 | GTCAAGTCCCTGAAGCCTGA | 60.386 | 20 | 55 |
| REV_1 | 700 | 1 | 756 | CGCATCCTGGTAAGAAGATT | 56.909 | 20 | 52.2 |
| REV_2 | 1400 | 701 | 1442 | TCCACCTCAGCACGTACATC | 59.707 | 20 | 55 |
| REV_3 | 2100 | 1401 | 2156 | CTTCCAAGTAACTCAGGGCG | 59.869 | 20 | 55 |
| REV_4 | 2800 | 2101 | 2844 | TTCTCGTGGTAGAGGTTCCG | 60.246 | 20 | 55 |
| brd3 | forward | 6002298 | 6002318 | AAGCAACAGATGCCTCCCTC | 60.03 | 20 | 55 |
| brd3 | reverse | 6002779 | 6002799 | CCGCTTAGCTGCTTGCTTTT | 59.76 | 20 | 50 |
| DDR | forward | 88342294 | 88342314 | CAGCATAGGGTACCAAGCCC | 60.18 | 20 | 60 |
| DDR2 | reverse | 88342867 | 88342891 | CATGAATGAGGAACAAAGTGTCCA | 59.48 | 24 | 41.67 |
| Fat1 | forward | 50398179 | 50398202 | AGCATCCTTAGCCATTAAACGGA | 60.12 | 23 | 43.48 |
| Fat1 | reverse | 50398634 | 50398655 | AGTACTCCAGCCTGTCTAGGT | 60 | 21 | 52.38 |
| Ncor2 | forward | 37032405 | 37032425 | GGTCCTGAGGTGGCTACTTG | 60 | 20 | 60 |
| Ncor2 | reverse | 37032852 | 37032872 | GATGCCAGCTCTGGATGGAC | 60 | 20 | 60 |
| Nono | forward | 71341038 | 71341061 | CCTGTAGTGGGAAGACCTTTGAG | 60.31 | 23 | 52.17 |
| Nono | reverse | 71341526 | 71341549 | ACCAGACAGACTTTAACGCACTA | 59.68 | 23 | 43.48 |
| Stag1 | forward | 109202303 | 109202326 | GGCATATGGACAGTATTTCATGG | 57.37 | 23 | 43.48 |
| Stag1 | reverse | 109202963 | 109202985 | TCCACTCTCAGCCACTTCTACA | 60.49 | 22 | 50 |
| Thrap3 (1) | forward | 144180643 | 144180666 | TCCATAAACTGACTTAGCCCTCC | 59.55 | 23 | 47.83 |
| Thrap3 (1) | reverse | 144181150 | 144181172 | TGGGAGACAACTTTCCATTCCC | 60.23 | 22 | 50 |
| Thrap3 (2) | forward | 144180693 | 144180716 | TCCATAAACTGACTTAGCCCTCC | 59.55 | 23 | 47.83 |
| Thrap3 (2) | reverse | 144181150 | 144181172 | TGGGAGACAACTTTCCATTCCC | 60.23 | 22 | 50 |
| Thrap3 (3) | forward | 144180715 | 144180738 | TCCATAAACTGACTTAGCCCTCC | 59.55 | 23 | 47.83 |
| Thrap3 (3) | reverse | 144181160 | 144181182 | TGGGAGACAACTTTCCATTCCC | 60.23 | 22 | 50 |


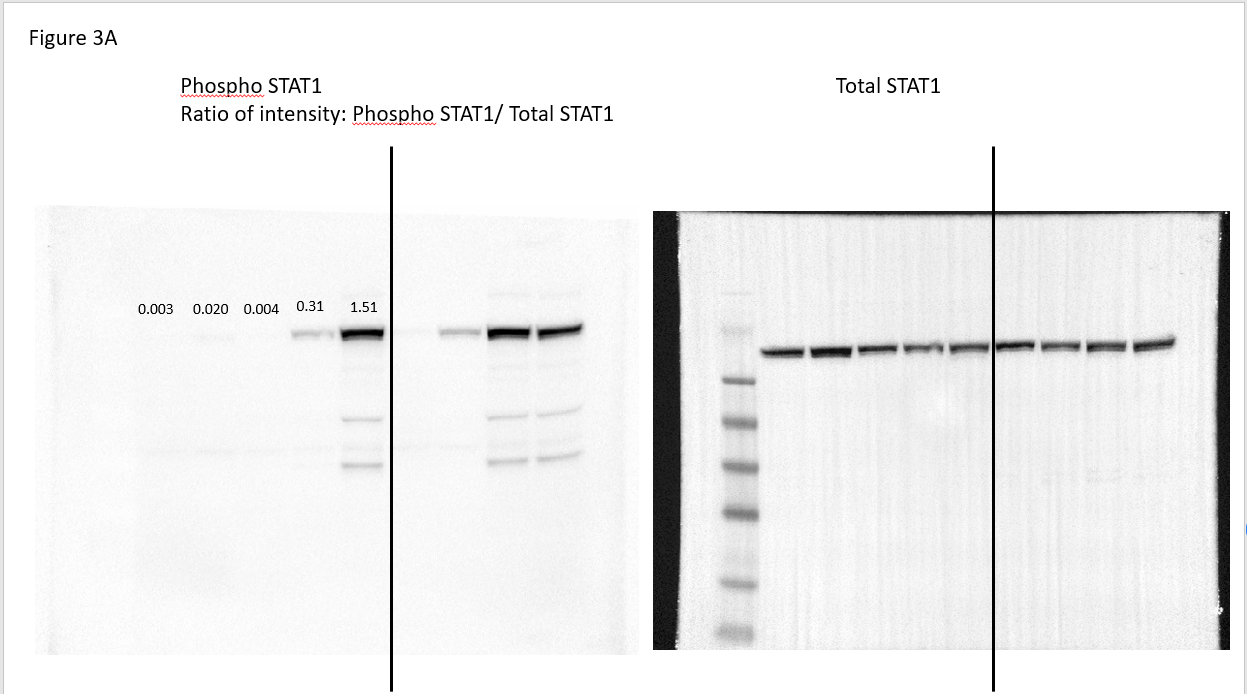


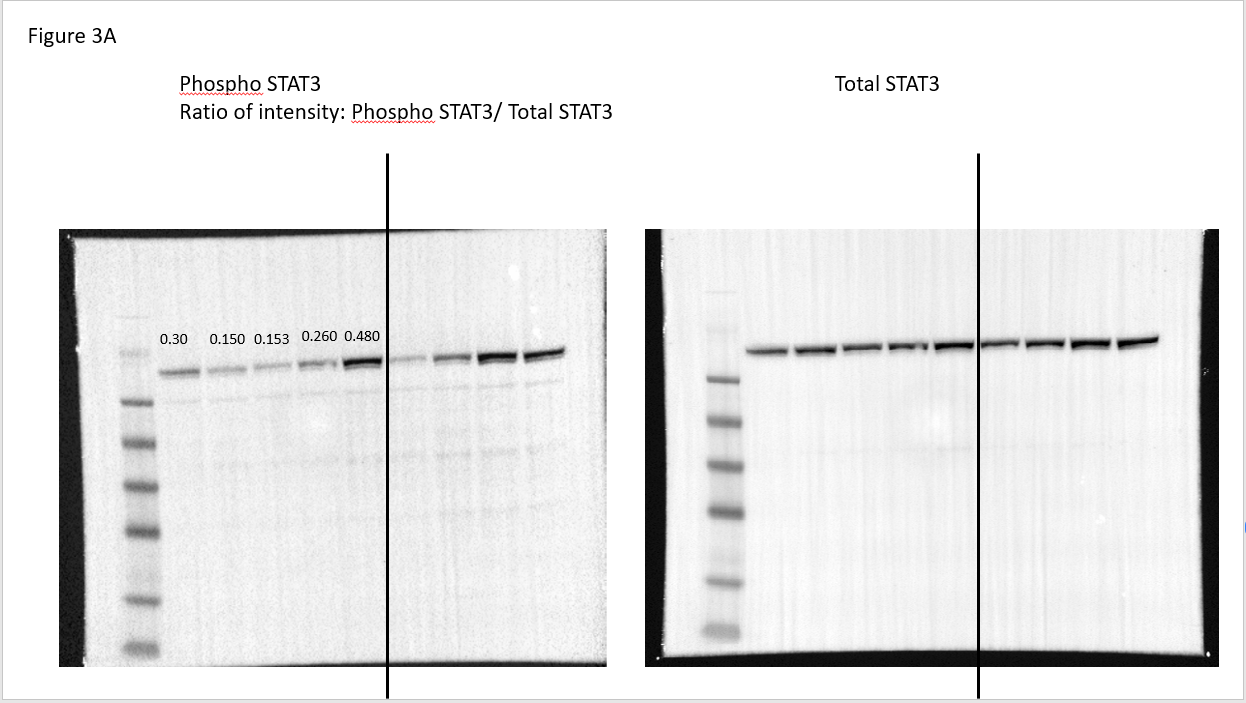


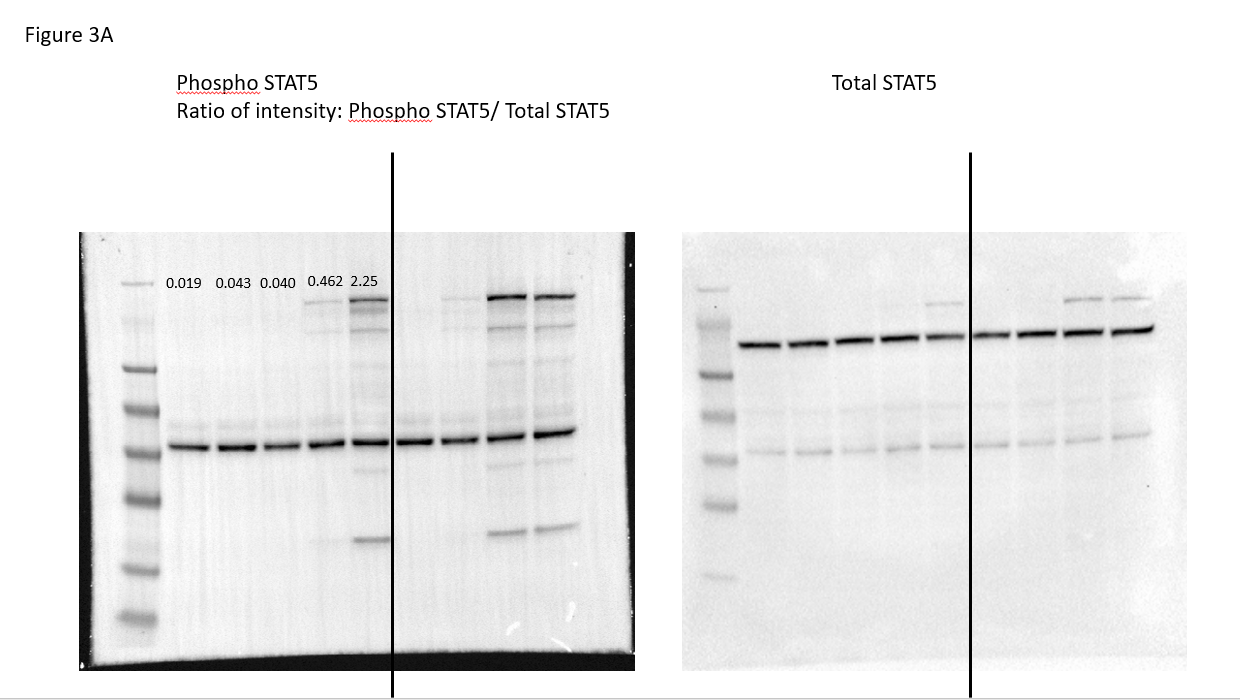


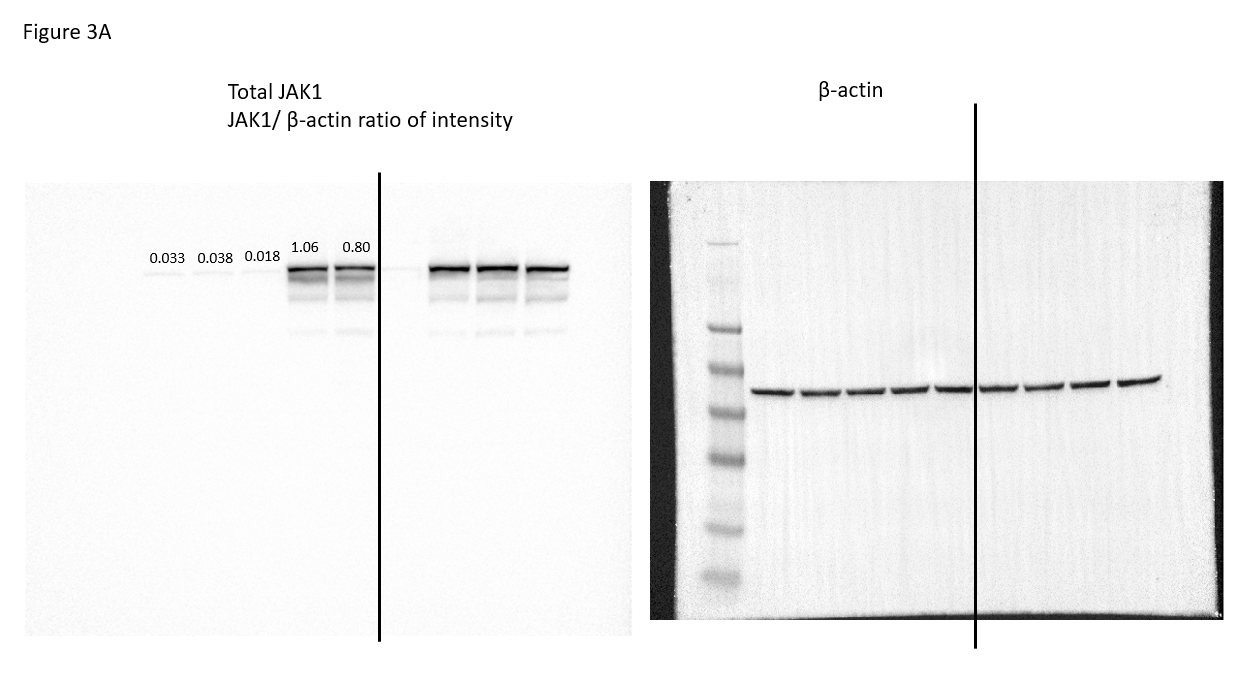


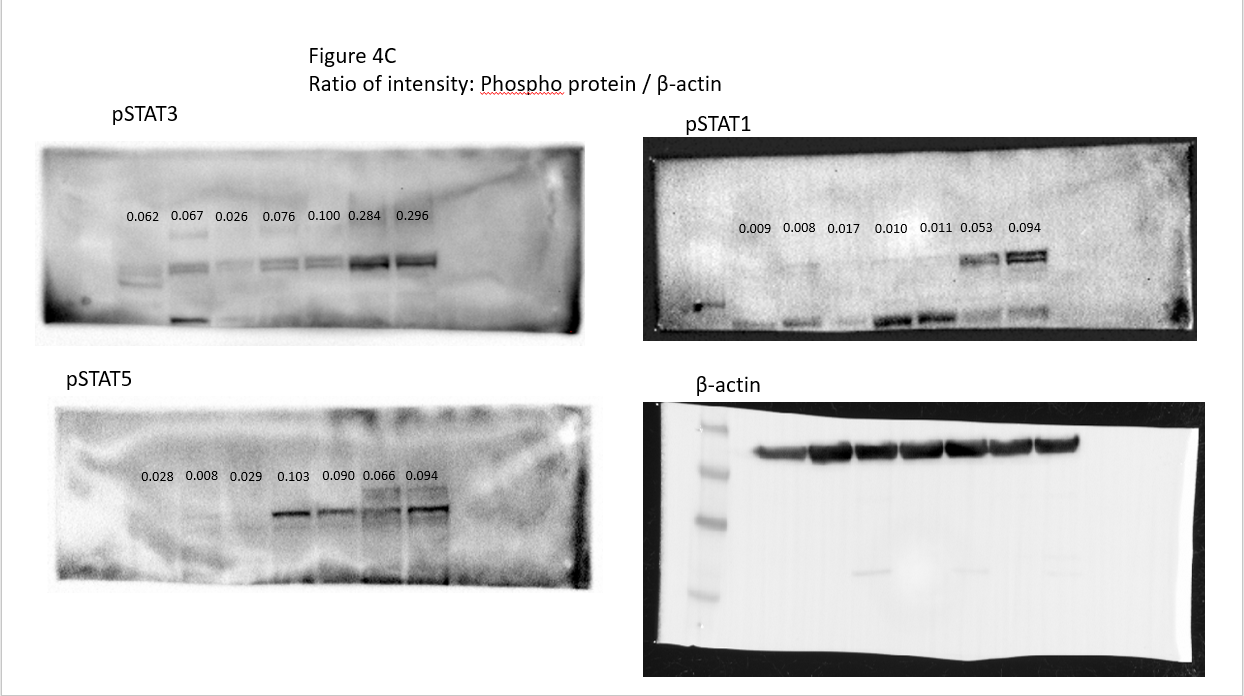


**Details of western blot.**

| 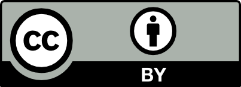 | © 2019 by the authors. Licensee MDPI, Basel, Switzerland. This article is an open access article distributed under the terms and conditions of the Creative Commons Attribution (CC BY) license (http://creativecommons.org/licenses/by/4.0/). |
| --- | --- |
